# The Use of Age Assessment in the Context of Child Migration: Imprecise, Inaccurate, Inconclusive and Endangers Children’s Rights

**DOI:** 10.3390/children6070085

**Published:** 2019-07-23

**Authors:** Ranit Mishori

**Affiliations:** Department of Family Medicine, Georgetown University School of Medicine, Washington, DC 20007, USA; mishorir@georgetown.edu

**Keywords:** age assessments, migration, child-protection, medico-legal ethics, forensic evaluations

## Abstract

Anecdotal reports suggest migrant children at the US border have had to undergo age assessment procedures to prove to immigration officials they qualify for special protections afforded to those under age 18. There are a variety of methods to assess the chronological ages of minors, including imaging studies such as X-rays of the wrist, teeth, or collarbone. However, these procedures have come under great scrutiny for being arbitrary and inaccurate, with a significant margin of error, because they are generally based on reference materials that do not take into account ethnicity, nutritional status, disease, and developmental history, considerations which are especially relevant for individuals coming from conflict and/or resource-constrained environments. Using these procedures for migration purposes represent an unethical use of science and medicine, which can potentially deprive minors with the protections that they are owed under US and international laws, and which may have devastating consequences. We should advocate for the creation special protocols, educate law enforcement and legal actors, ensure such procedures are carried out only as a last resort and by independent actors, emphasize child protection and always put the child’s best interest at the core.

## 1. Introduction

A 17-year-old teenage boy fleeing gang violence in Honduras was apprehended by the U.S. Border Patrol in Texas and placed in detention in Arizona. Though he informed detention staff of his age, they accused him of lying and placed him in an adult facility, where adult detainees threatened and harassed him over his $5.00 telephone card. He once again told the authorities that he did not belong in the adult facility. They said: Prove it. Detention staff took him to the prison clinic for a dental exam, which was interpreted to indicate that he was 16–17 years old, so he was subsequently transferred to an age-appropriate facility. He was evaluated by a clinical psychologist who conducted a forensic evaluation for his asylum case, documenting psychiatric symptoms and psychological effects of the gang violence he had experienced. His asylum case is still pending.

This story seems like a positive example of science and medicine in service of child protection. However, the dental assessment, with its two-year margin of error range, could easily have gone the other way, and caused an adolescent to be inappropriately placed in an adult facility, without the procedural and substantive protections due to minors under U.S. and international law.

## 2. What Are Age Assessments?

Age assessments generally refer to any procedures used to establish an individual’s chronological age—the age from the day that person was born. Chronological age is, in and of itself, of limited value in predicting maturity, social or intellectual ability, or a person’s capacity to function in a new environment.

There are a variety of methods to assess the chronological ages of minors [1]. From history taking, to a medical examination (physical exam, sexual maturity); the use of official state documents; Anthropometric evaluation (measurements of height, weight, head circumference); imaging studies—X-rays of the wrist, teeth, or collarbone, or MRIs [2]. Table 1 offers a review of these methods and associated information.

As a clinical procedure, skeletal age assessments are used frequently in pediatric endocrinology [10]. Dental age assessments have been used, among other things, for international adoptions [11]. These radiographs of the dental crown and root of the third molar tooth are compared with reference studies to determine age. Similarly, hand and wrist radiographs are compared to radiographs from reference studies in order to judge skeletal age and bone maturation. However, multiple studies have concluded that both methods are only able to produce estimates within a range of at least two years. Research studies have demonstrated that these methods systematically under and over estimate ages [12,13,14].

Imaging tests have come under great scrutiny for being arbitrary and inaccurate, with a significant margin of error [12,13]. This is mostly because they are generally based on reference materials that do not take into account ethnicity, nutritional status, disease, and developmental history, considerations that are especially relevant for individuals coming from conflict and/or resource-constrained environments. Other concerns are that these tests are invasive, expensive, and potentially harmful exposing minors to unnecessary radiation.

These inaccurate procedures represent an unethical and unprofessional use of science and medicine for procedures that are both inconclusive and can potentially deprive those under the age of 18 with the protections that they are owed under the US and international human rights laws. Inaccurate assessments may have devastating consequences for children who may suddenly be ‘determined’ to be an adult, thus denied special protection and other human rights provisions. Such protections usually include protection from abuse, abandonment, and neglect and codified requirements to promote children’s safety, education, health, and nutrition, and protect them from exploitation and abuse [15,16].

For example, being moved to an adult detention center without appropriate services for minors and where their safety may be at risk is counter to child-protection provisions. Other examples under US law include loss of the right to non-adversarial asylum proceedings, limits on duration of detention, and support for reunification with parents and other relatives [15,16].

## 3. What Can We Do?

There are no standardized protocols in the US meant to offer guidance regarding the use of age assessment methods for minors. It is imperative that they be developed with input from physicians, social workers, human rights experts, and other stakeholders who can review possible scenarios through a child-protection lens.

As clinicians and as human rights defenders, we must first acknowledge, and ensure others are aware, that the determination of the chronological age of a child is almost never accurate or precise. It is not an exact science. In the context of migration (as opposed to pediatric endocrinology, for example) there are significant social consequences and potential risk to the safety of the minor whose age is being assessed or disputed. While in some medical contexts there is merit to such testing, in legal contexts it is a more dubious practice. Therefore, age assessments for migration purposes, especially via radiologic imaging, should be carried out only as a last resort.

Key considerations must be given to who should have a mandate to request these tests, and for what reasons. Motivation and reasoning may vary based on the requesting entity: Border patrol, governmental agents, representatives of the judicial system handing asylum cases, social workers, and physicians. Not all of these stakeholders may have the child’s best interest at heart, or the intention and means of ensuring child protection above all.

If or when age assessment procedures are ordered, they must be carried out by independent professionals and those who have expertise in performing them and interpreting them appropriately; by professionals who are familiar with genetic, medical, and ethnic variations, and differences in cultural background. We should strive to create protocols that specify (or even restrict) who is allowed to refer minors for or order age assessments.

When considering whether to subject a child to an age assessment, evaluators should balance physical, developmental, psychological, cultural, and environmental factors. They must never force such assessments on minors, must avoid invasive or intrusive exams and must always choose the least invasive assessment first.

Consent protocols should be followed, and informed consent must be obtained every time for these procedures in accordance with common pediatric guidelines [17]. Protections must be developed to ensure that minors are never forced, coerced, or pressured to undergo age assessments, and every effort must be made to ensure that a child’s dignity is preserved. We must also strive to create and implement safeguards to address appeals in cases of disputed results. If a child refuses to undergo any kind of age assessment, it should not be held against him/her, or prejudice the assessment or protection measures.

Ultimately, our goal should be to reduce the use of such exams and use them only as a last resort. Individually and as a profession, the best interest of the child must always be our guiding principle, and we must holistically assess each child’s vulnerability and unique needs, in line with international guidelines [13,18].

## 4. Use Our Collective Voices

We should also urge professional medical organizations and associations such as the American Academy of Pediatrics, American Dental Association, American College of Dentists, American Board of Forensic Odontology, American College of Radiology, and the American Academy of Forensic Sciences to offer guidance to their members about all the medical, legal, and ethical issues inherent in age assessments and to help educate other stakeholders—for example, immigration judges—about common pitfalls of using imaging for age assessment.

## Figures and Tables

**Table 1:** Different methods for age assessments.

Non Medical	How It Is Done; by Whom	Possible Issues
Documentation	Retrieve, review, and request documents such as a birth certificate, immunization record, or others that might have the child’s DOB.	Many children will not have any papers; papers get lost. There are no standards on documentation of identity or age. Contacting family members in the home country may be an option in trying to retrieve official state documents.
Interview and history taking	Use the history and patient/client narrative, or any family member to try to assess the year of birth of the child in question.	Avoid an intimidating style of interview. Allow only professionals with training to elicit this information. There are no specific protocols on how to conduct such interviews.
Medical No one should rely on physical appearance to determine a child’s chronological age, as there are significant variations in physical development.		
Physical—sexual maturity	Use standard protocols for sexual maturity assessment such as Taner Staging [3].	Taner staging may be less useful in late adolescence and in those with an early onset of puberty. Visual inspections can be traumatic to children, especially those who may have experienced sexual violence. Never take pictures without adhering to thorough consent processes.
Physical—anthropomorphic	Use height, weight, skin rating, and compare to reference values [4,5].	Such measurements often do not reflect variations due to race, ethnicity, nutritional status, and socio-economic status.
Imaging Studies They rely on skeletal changes that occur as children’s bones mature; significant bias exists in interpretation and such imaging studies can never report the precise chronological age of a child. Variations range is generally accepted to be +2/−2 years [6].		
Radiological Tests—(carpal) hand and wrist X-rays	Assess the fusing progression of carpal bones.	The most common method used. Data relies on populations samples that do not reflect diversity of race, ethnicity, nutritional status, and SE background. There are no standards for specific populations (Latino, African, and Middle Eastern). Radiation exposure.
Radiology Dental X-rays	Relies on presence, absence, or development of the roots of the 3rd molars.	Data relies on populations samples that do not reflect diversity of race, ethnicity, nutritional status, and SE background. There are no standards for specific populations. Radiation exposure.
Radiology: Collar bone X-rays	Assesses the fusing process of the clavicle.	Data relies on populations samples that do not reflect diversity of race, ethnicity, nutritional status, and SE background. There are no standards for specific populations. Radiation exposure.
MRI of the knee or hand [7,8]	It has been suggested as a method to counter the ethical problems with X-ray use and avoid radiation [8].	Attracting increasing attention. Concerns for incidental findings and follow up. More expensive.

(Adapted from the Position Paper on Age Assessment in the Context of Separated Children in Europe 2012) [9].
